# Personalized medicine and nutrition in hepatology for preventing chronic liver disease in Mexico

**DOI:** 10.3389/fnut.2024.1379364

**Published:** 2024-05-09

**Authors:** Arturo Panduro, Sonia Roman, Irene M. Mariscal-Martinez, Alexis Jose-Abrego, Karina Gonzalez-Aldaco, Claudia Ojeda-Granados, Omar Ramos-Lopez, Luis A. Torres-Reyes

**Affiliations:** ^1^Department of Genomic Medicine in Hepatology, Civil Hospital of Guadalajara, Fray Antonio Alcalde, Health Sciences Center, University of Guadalajara, Guadalajara, Jalisco, Mexico; ^2^Department of Medical and Surgical Sciences and Advanced Technologies “GF Ingrassia”, University of Catania, Catania, Italy; ^3^Medicine and Psychology School, Autonomous University of Baja California, Tijuana, Baja California, Mexico

**Keywords:** Mexico, genetics, diet-related adaptive genes, hepatopathogenic diet, Genomex diet, nutrients, training, genomic medicine in hepatology

## Abstract

Chronic liver disease is a global health issue. Patients with chronic liver disease require a fresh approach that focuses on the genetic and environmental factors that contribute to disease initiation and progression. Emerging knowledge in the fields of Genomic Medicine and Genomic Nutrition demonstrates differences between countries in terms of genetics and lifestyle risk factors such as diet, physical activity, and mental health in chronic liver disease, which serves as the foundation for the implementation of Personalized Medicine and Nutrition (PerMed-Nut) strategies. Most of the world’s populations have descended from various ethnic groupings. Mexico’s population has a tripartite ancestral background, consisting of Amerindian, European, and African lineages, which is common across Latin America’s regional countries. The purpose of this review is to discuss the genetic and environmental components that could be incorporated into a PerMed-Nut model for metabolic-associated liver disease, viral hepatitis B and C, and hepatocellular carcinoma in Mexico. Additionally, the implementation of the PerMed-Nut approach will require updated medicine and nutrition education curricula. Training and equipping future health professionals and researchers with new clinical and investigative abilities focused on preventing liver illnesses in the field of genomic hepatology globally is a vision that clinicians and nutritionists should be concerned about.

## 1 Introduction

**Figure 1:**
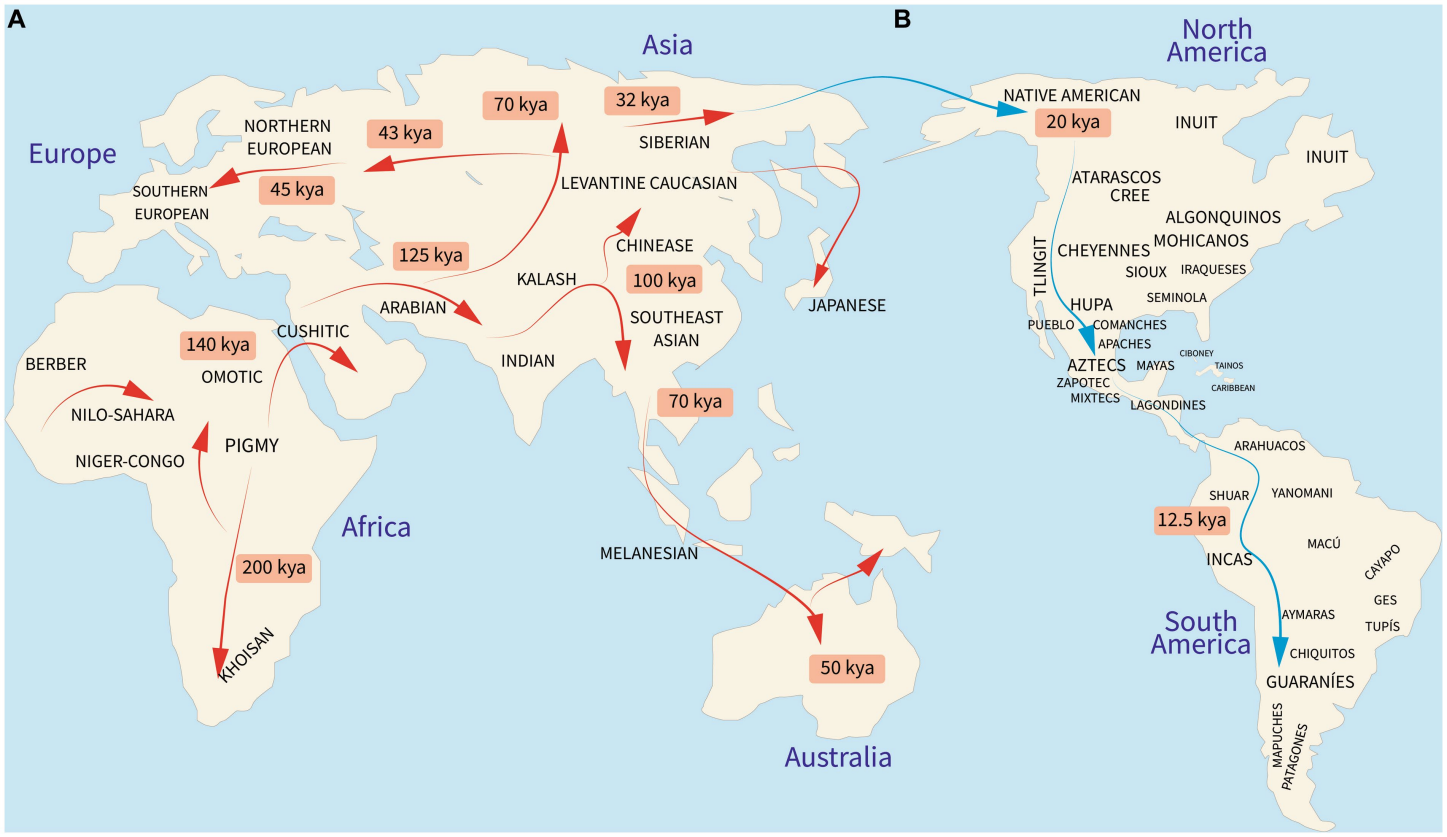
Peopling of the world. **(A)** Migration of the 19 ancestral human populations from which Native Americans made their way to the Americas. **(B)** Peopling and main settlements of the Americas in pre-Colombian times by the ancestral Native American component. kya, kilo years ago. Reproduced from references (1–3).

Modern humans, taxonomically known as *Homo sapiens sapiens*, emerged in Africa 200,000 years ago and began the migration out-of-Africa to colonize the globe between 50,000 and 70,000 years ago (1, 2). Studies on human evolutionary history relate that most of the world’s populations have descended from 19 ancestral human ethnic groups (Figures 1A,B) (2, 4). However, the human genome, which is constantly evolving, is a melting pot of past, present, and even future genetic signs of adaptations to diverse environmental niches, and it appears that they are not always meant to create life-threatening disorders (5, 6). These differences are explained by genetic variants including single nucleotide polymorphisms (SNPs), copy number variations (CNVs), and deletion or insertion (Del/Ins) polymorphisms (7). Other non-genetic mechanisms, such as transgenerational plasticity and cultural evolution, were undoubtedly additional powerful forces that contributed to human adaptation (8). However, discrete changes in the nature and frequency of specific genetic variants might influence health triggered by environmental factors (9). Such variants have been revealed with the completion of the Human Genome Project’s mapping of the human haploid reference genome (10) and the incorporation of other worldwide genomic databases (11) so that the understanding of the genetic basis of human disease and, ultimately, the prevention of many diseases may be achieved (12).

Currently, the paradigm shift in medicine from reactive to predictive is focused in providing models of prevention strategies based on the population’s genetic makeup and the risk factors that contribute to the emergence of chronic diseases, whether infectious or non-communicable (13). Clinicians need to reconsider their traditional approach to managing major diseases (such as hypertension, heart disease, type 2 diabetes, cancer, renal, and liver disease) from a bench to bedside/translational perspective, combining novel omics data with pathological clinical phenotypes in search of an individualized therapy (14). This has resulted in a multitude of terms, including Predictive Medicine, Individualized Medicine, Personalized Medicine, and Precision Medicine (15, 16), all of which acknowledge that each person is unique but still shares ancestral genetic variants based on ethnicity and environmental circumstances. These characteristics may explain why certain people are susceptible or prone to diseases when exposed to risky conditions or behaviors, but others in similar circumstances (i.e., family members) are unaffected (17). Personalized Medicine “omics” techniques have been proposed for nearly all medical fields including Nutrition, where Genomic Nutrition, Personalized Nutrition, and Precision Nutrition are becoming increasingly important (18).

The Personalized Medicine and Nutrition (PerMed-Nut) approach in Hepatology will inevitably include managing the main etiologies that cause chronic liver disease worldwide (19), such as alcohol persistence, chronic viral hepatitis B and C infections, metabolic-associated steatotic liver disease, hepatotoxicity (drugs or toxins), and auto-immune conditions. However, the prevalence of these etiologies varies greatly between countries due to genetic, environmental, and cultural differences in the host populations (20). As a result, the extent of liver damage might be immediate and self-limiting, or it can progress to chronic phases in which the inflammatory process of injury causes fibrosis, cirrhosis, and, in rare cases, hepatocellular carcinoma (HCC).

How can we begin using the PerMed-Nut approach as a strategy to prevent chronic liver diseases? Firstly, it is essential to understand the impact of genes and the environment at a regional level to develop measures to prevent liver damage. Hence, this work aims to define these factors for the prevention and management of liver illnesses using a PerMed-Nut model and the need for training in genomic education. We will address some polymorphic genes associated with the clinical outcomes of liver diseases as well as sociocultural features of the Mexican population in the sections that follow.

## 2 Mexico’s genetic ancestry and sociodemographic background

Studies examining the genetic ancestry of the Latin American (LATAM) populations including Mexico have revealed heterogeneous proportions of three main ancestral lineages: Amerindian, European, and African. As shown in Table 1, most LATAM countries contain all three with some having a higher degree of African (21, 28) and Native American (22, 27, 29, 30) ancestry than European (3, 23–26, 29). The indigenous people of Mexico are part of the third Native American migration wave that crossed the Bering Strait to the American continent around 20,000 years ago (2) (Figure 1B, blue arrow). These Native Americans settled primarily in Mexico (Tenochtitlan) and Peru (Cuzco) followed by Brazil, the United States, Bolivia, Columbia, and in lesser proportions Argentina, Venezuela, Ecuador, Canada, and Alaska (31). The current 68 ethnic groups of Mexican Amerindians are descendants of the original First Nations who followed either a nomadic or agricultural lifestyle, primarily in Aridoamerica and Mesoamerica, respectively (3, 32). During the Spanish colony (from 1521 to 1826), a genetic and cultural admixture occurred among Mexican Amerindians, Spaniards, and enslaved Africans (21). The Spanish Catholic Church recognized new births using a caste system, with Amerindian and Black heritage overlooked that has marked the social distribution of the ancestral lineages since colonial up until modern times.

**Table 1:** Distribution of the ancestral components among LATAM populations.

Country/(Reference)	GAM	Ancestry (%)			
		Native American	European	African	Asian
Argentina (3)	AIM	27.7–32.3	65.6–68.4	2–3.8	-
Barbados^*^ (21)	Y-DNA	1	-	87	-
Bolivia (22)	AIM	77–86	13–21	2	-
Brazil (23)	AIM	17.3	59.7	23	-
Chile (24)	SNP	43	55	2	-
Colombia (25)	CODIS	25.22	64.75	9.01	1.03
Cuba (26)	mtDNA	4	70	26	-
Dominican Republic (26)	mtDNA	6	56	38	-
Ecuador (27)	HLA	53–63	29–47	19	-
Haiti (28)	STR	0	4	96	-
Mexico (24)	SNP	51	46	3	-
Mexico (25)	CODIS	44.52	43.26	4.8	4.45
Paraguay (29)	mtDNA	33.8	55.4	10.8	-
Peru (29)	CODIS	73.76	20.36	3.01	2.87
Puerto Rico (29)	CODIS	12.82	71.5	15.01	0.67
Uruguay (30)	SNP	27	30–70	7	-

GAM, Genetic ancestry marker. ^*^Afroamericans; Y-DNA, Y chromosome inheritance; AIM, Ancestry informative marker; SNP, Single nucleotide polymorphisms; CODIS, Combined DNA index system; mtDNA, Mitochondrial DNA; HLA, Human leukocyte antigens; and STR, Short tandem repeats.

Most Mexicans are admixed containing practically half of the main lineages (24, 25). However, as illustrated in Table 2, the degree of admixture varies across the country. Northern Mexico has a higher rate of European trait carriers (33, 34) and intermediate rates in the Central Region (35–37), contrasting with Southern Mexico, who are more likely to be carriers of Amerindian traits (35, 39) including the true Native Americans *per se* (38). African ancestry is present in varying quantities among the admixed general population and Afro-Mexicans who reside along the South Pacific and the Gulf of Mexico coastal communities (40). Further genetic studies show indigenous mitochondrial DNA as the maternal source of inheritance, while Y-STR analysis tracks European paternal lineage (37, 41).

**Table 2:** Distribution of the Mexican ancestral lineages throughout the country.

Region/Reference	GAM	Ancestry (%)			
		Native American	European	African	Asian
**North**					
Chihuahua (33)	STR	38	50	12	-
Northeast (34)	AIM	38	56	6	-
Nuevo Leon (34)	AIM	40	55	5	-
**Central**					
Jalisco (35)	STR	53	31	16	-
Zacatecas (36)	AIM	51	46	3	-
Mexico City (37)	Y-DNA	66.6	23.3	6.6	3.5
**South**					
Yucatan (35)	STR	70	19	11	-
Puebla (35)	STR	72	17	11	-
Guerrero (35)	AIM	95	4	1	-
**Native Amerindian**					
Yaquis (38)	AIM	72	28	-	-
Huicholes (38)	AIM	92	8	-	-
Seris (38)	AIM	92	8	-	-
Teenek (38)	AIM	98	2	-	-
Zapoteco (38)	AIM	98.1	1.9	-	-

GAM, Genetic ancestry marker; Y-DNA, Y chromosome inheritance; AIM, Ancestry informative marker DNA; and STR, Short tandem repeats.

**Figure 2:**
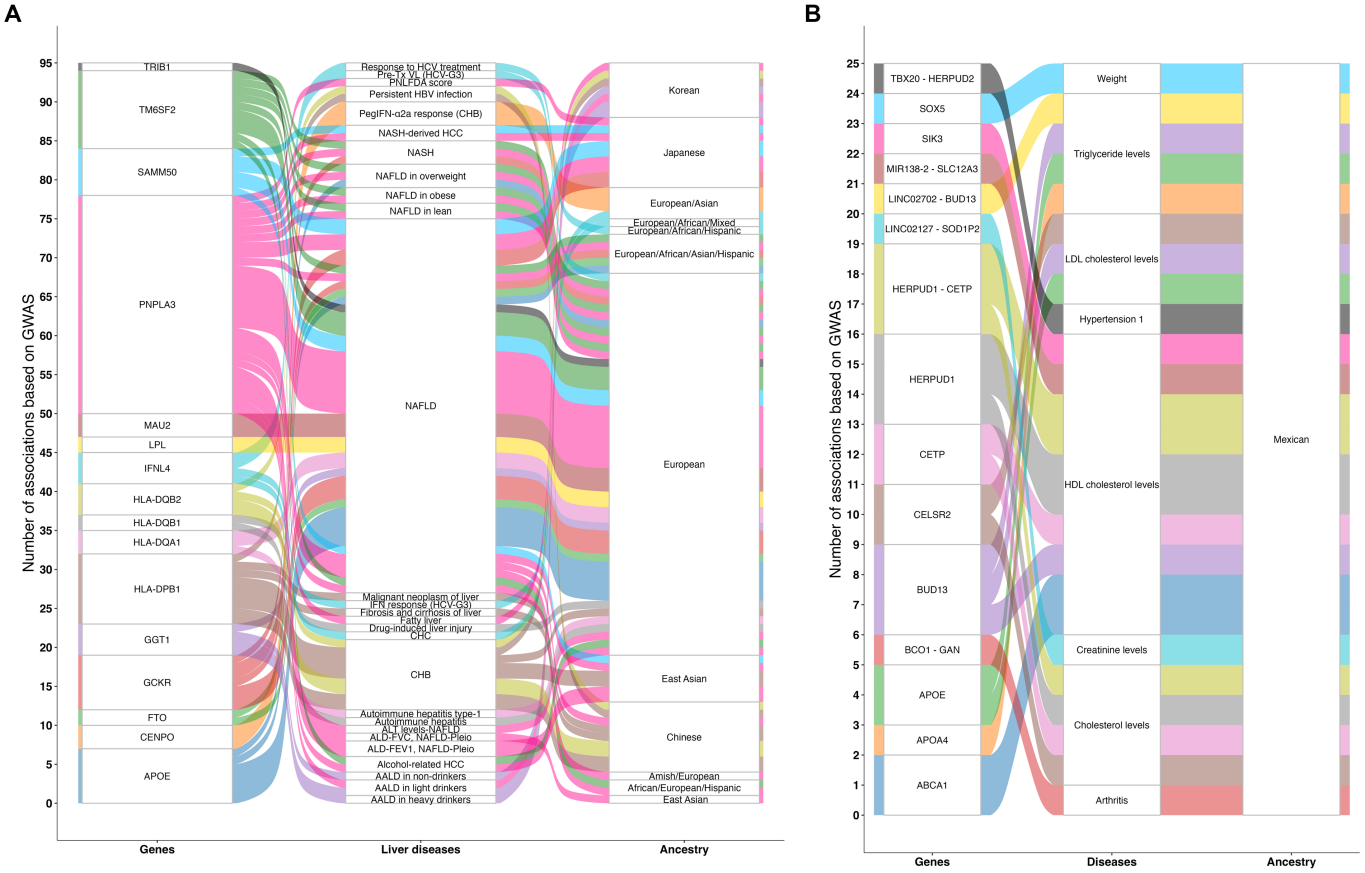
Genomic studies related to liver diseases. **(A)** Illustration of 284 GWAS related to liver diseases that have been conducted, identifying 1,546 associations in over 606 genes that involve the regulation of body weight and fat mass, glucose metabolism, peptide metabolism, lipid metabolism and transport, centromere structure and function, immune system, response to viral infections, meiotic cell division, mitochondrial membrane protein assembly, and cellular proliferation. **(B)** Illustration of the results of the Mexican Biobank analysis showing 25 associations in 15 genes involved in lipid metabolism and transport, vitamin A metabolism, protein folding, regulation of gene expression, cellular signaling, embryonic development, and heart development. Among the most important clinical associations are hypercholesterolemia, hyper triglyceridemia, body weight, creatinine levels, hypertension, and arthritis. The lines on the chart indicate the number of associations found between each gene, disease, and ancestral group. GWAS, Genome-wide association study; ALD-FEV1, NAFLD-Pleio, Accelerated lung function decline (FEV1) and worsened/persistent change in fatty liver (pleiotropy); ALD-FVC, NAFLD-Pleio, Accelerated lung function decline (FVC) and worsened/persistent change in fatty liver (pleiotropy); ALT levels-NAFLD, Alanine aminotransferase levels in non-alcoholic fatty liver disease; AALD in heavy drinkers, Alcohol-associated liver disease in heavy drinkers; AALD in light drinkers, Alcohol-associated liver disease in light drinkers; AALD in non-drinkers, Alcohol-associated liver disease in non-drinkers; Alcohol-related HCC, Alcohol-related hepatocellular carcinoma; Autoimmune hepatitis, Autoimmune hepatitis in primary sclerosing cholangitis; Autoimmune hepatitis type-1, Autoimmune hepatitis type-1; CHB, Chronic hepatitis B infection; CHC, Chronic hepatitis C infection; Drug-induced liver injury, Drug-induced liver injury (amoxicillin-clavulanate); Fatty liver, Fatty liver; Malignant neoplasm of liver: ICD10 C22, Malignant neoplasm of liver and intrahepatic bile ducts; Fibrosis and cirrhosis of liver: ICD10 K74, Fibrosis and cirrhosis of liver; NAFLD, Nonalcoholic fatty liver disease; NASH, Nonalcoholic steatohepatitis; NASH-derived HCC, Nonalcoholic steatohepatitis-derived hepatocellular carcinoma; PNLFDA score, Pediatric non-alcoholic fatty liver disease activity score; PegIFN-α2a response (CHB), Peginterferon alfa-2a treatment response in chronic hepatitis B infection; Persistent HBV infection, Persistent hepatitis B virus infection; Pre-Tx VL (HCV-G3), Pre-treatment viral load in hepatitis C virus genotype 3; Response to HCV treatment, Response to hepatitis C treatment; IFN response (HCV-G3), Response to interferon treatment in hepatitis C virus genotype 3. Source: GWAS catalog liver diseases, licensed under CC0. Summary statistics were downloaded on March 11, 2024 from the NHGRI-EBI GWAS Catalog. Accession ID: EFO_0001421.

Among the Genome-Wide Association Study (GWAS) database catalog, a total of 284 studies related to liver diseases have been conducted, identifying 1,546 associations in over 606 genes (42, 43). They are mainly reported in European, Asian, and African populations and scarcely in Native American ancestry (Figure 2A). However, one GWAS using the Mexican Biobank have shown 25 associations in 15 genes involved in lipid metabolism and transportation, vitamin A metabolism, protein folding, regulation of gene expression, cellular signaling, embryonic development, and heart development (Figure 2B). Among the most important clinical associations are hypercholesterolemia, hyper triglyceridemia, body weight, creatinine levels, hypertension, and arthritis. Furthermore, this study confirmed earlier findings of the north–south gradient of European-Amerindian ancestry (44).

**Figure 3:**
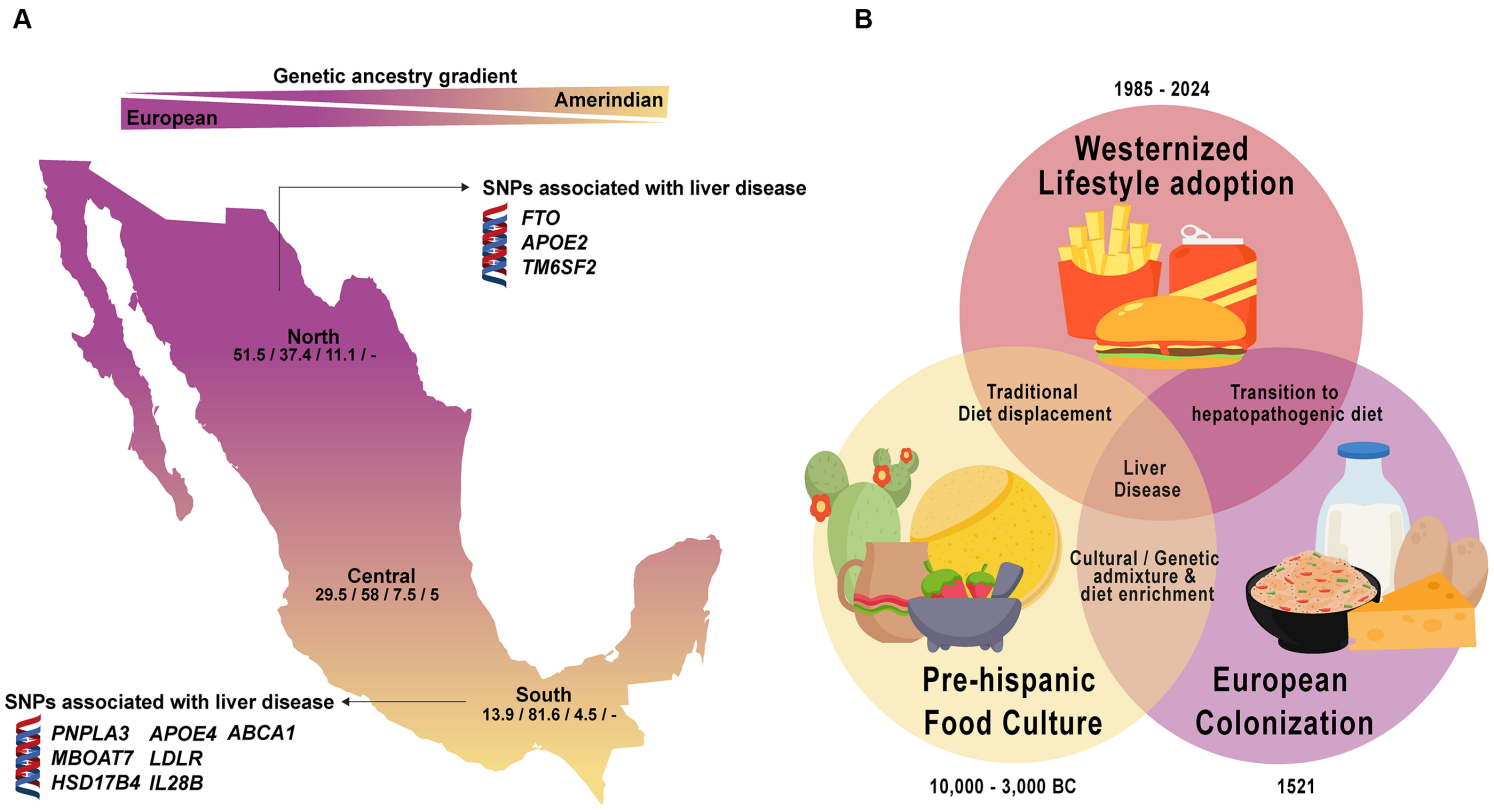
Ancestral genetic gradient and nutrition transition in Mexico. **(A)** Contemporary Mexicans show a tripartite ancestry admixture with predominant European ancestry in the North and increasing Amerindian ancestry towards the southern region. Genetic variants linked to liver disease vary based on population ancestry. Numbers in the map represent the average percentage of the European/Amerindian/African/Asian component. **(B)** Dietary transition in Mexico reflects a shift from traditional to a westernized hepatopathogenic diet, contributing to a higher liver disease prevalence in the population.

Overall, the underlying genetic heterogeneity of most LATAM populations including Mexico constitutes a key aspect that makes sub-structure analysis and GWAS combined important tools to reveal which lineage is carrying the disease-related genetic polymorphisms and, consequently, the genetic foundation of disease vulnerability (17, 45). As shown in Figure 3A, regional variations in the ancestral component carrying the genetic variants as well the interactions with environmental and lifestyle factors support the need of regional PerMed-Nut strategies to address these differences (46).

## 3 Environmental changes and evolutionary genetic mismatch

One important factor in the gene–environment interaction equation is diet. The domestication of endemic wild plants and animals by the prehispanic groups of ancient Mexico, approximately 6,000 years ago, is a historic milestone for the peopling and development of the Aztec civilization and other ethnic groups because it provided the primary source of nutrients for the indigenous communities (47). Mexico is noted for three staple plants: maize, beans, and chili, along with squash, tomato, amaranth, and chia growing in an eco-agricultural style known as “milpa” using the “chinampas” system (48). The inverted Mesoamerican food pyramid contains edible leafy green vegetables and wild fruits at the top, cereals/grains at the middle, and animal meat at the bottom, implying that it was consumed in smaller quantities because animal rearing was uncommon. The exposure to this diet over millennia may have exerted positive selective pressure on the Mexican genome, favoring the dominance of diet-related adaptive gene (DRAG) polymorphisms that regulate essential energy, immunological, and nutritional pathways (47). Based on the earlier environmental conditions, the adaptive allele, which was selected over the wild allele, is a risk allele for disease in unfavorable conditions such as the occurring globalized nutrition transition (48–50). Different allelic frequencies of these DRAGs may influence the risk of complex diseases among populations based on interactions with dietary contexts, such as the current obesity endemic. For example, the cholesterol transporter ATP-binding cassette transporter A1 (*ABCA1*) gene variant (R230C, rs9282541) is unique to Native American individuals and has been associated with low high-density lipoprotein cholesterol (HDL-C) levels, obesity and type 2 diabetes in admixed Mexicans (51, 52). The positive selection of the C allele among the Native American population may be related to energy-saving processes among the ancient Mesoamericans. Overall, the current frequency of the *ABCA1* 230RC + CC genotypes ranges from 6 to 20% (46) and studies performed in central west Mexico have shown that up to 41% of Native American individuals who are carriers of the *ABCA1* C allele have low HDL-c levels compared to admixed Mexicans in 7% (53), suggesting the need of targeted strategies among the population.

In this sense, populations that maintain their historical staple foods are less likely to develop nutrition-related disorders (54). However, when combined with imbalanced modern diets, these adaptive genes may generate pathological processes. In Mexico, globalization and urbanization have driven the nutrition transition, leading to a shift toward a hepatopathogenic diet containing ultra-processed foods, unhealthy dietary habits, and an imbalance in essential fatty acids, vitamins, and minerals (48, 55) (Figure 3B). Notably, a recent study analyzing data from the National Health and Nutrition Survey (2018–2019) revealed that 68% of the Mexican population consumes a western dietary pattern high in processed foods compared to only 7% of those following a traditional Mexican diet (56). Currently, 72.1% of the Mexican population is overweight or obese; this risk factor, in combination with genetic vulnerability, has increased the incidence of cardiovascular disease, type 2 diabetes, cerebrovascular events, hepatopathologies, and cancer (breast and prostate) (57). Thus, understanding the ancestral and historical transitions is the foundation for developing a regionalized PerMed-Nut approach to address the dynamic interplay between genetic and environmental features that could affect the outcome of liver diseases in this region.

## 4 Managing chronic liver disease using a PERMED-NUT approach

### 4.1 Metabolic-associated liver disease

Genetic susceptibility (Figure 2) and the consumption of an unhealthy diet are the two most important risk factors related to metabolic-associated steatotic liver disease (MASLD) (formerly known as non-alcoholic fatty liver disease, NAFLD), which encompasses fatty liver or steatosis and metabolic-associated steatohepatitis (MASH) (formerly non-alcoholic steatohepatitis, NASH). Hence, a PerMed-Nut approach for the management of MAFLD would require knowledge of the allele/genotype frequency of several genes involved in food preferences and lipid/carbohydrate metabolism.

Food preference genes may partially explain the risk of abnormal lipid parameters and liver damage among the Mexican population. In this regard, some candidate-gene studies have been performed one example is the A allele (rs1761667) of the class B scavenger (*CD36*) receptor gene, which was found to be associated with higher fat intake and hypercholesterolemia in overweight individuals and more instances of liver damage in chronic hepatitis C patients (58, 59). Moreover, Mexicans carrying the Val allele (rs35874116) of the sweet taste receptor (*TAS1R2*) gene consumed more carbohydrates and had increased serum triglyceride levels (60). Furthermore, the novel AVV haplotype of the bitter taste receptor (*TAS2R38*) gene was first identified in the Mexican population and was associated with high alcohol intake (61). However, because genome structure corrections were not performed in these studies, further research is required to confirm these results.

Apolipoprotein E (APO E) is a plasma protein that transfers lipids from circulating lipoproteins to tissues through binding membrane receptors (62). The *APOE* gene encodes three major protein isoforms, E2, E3, and E4 with differential binding receptor affinities (63). The frequency of the *APOE* alleles differs worldwide (64). Recently, we identified a differential distribution of lipid genes among several ethnic groups showing that the *E4* allele associated with hypercholesterolemia was more frequent in Mexican Amerindians, whereas those associated with hypertriglyceridemia such as *E2* are common among European populations ancestry (53).

Another functional gene is the Patatin-like phospholipase domain-containing protein 3 (*PNPLA3*) I148M (rs738409 C > G) variant, one of the most studied genetic determinants implicated in liver damage associated with the generation of a pro-inflammatory environment and oxidative stress (65, 66). This variant has shown a geographic relationship with the prevalence of MASH and it has been documented that the *PNPLA3* risk G allele (148 M) highly prevails in Mexican Amerindians compared to admixed populations (67). This variant has been strongly associated with elevated alanine transaminase (ALT) levels in normal weight and overweight/obese Mexican children suggesting that it may be a risk factor for liver damage (68). Furthermore, the Transmembrane 6 superfamily 2 (*TM6SF2*) gene is involved in plasma lipid regulation by influencing triglyceride secretion and hepatic lipid droplet content. The *TM6SF2* E167K (rs58542926) polymorphism has been associated with impaired hepatic lipid synthesis in NAFLD patients (69). Additionally, the fat mass and obesity (*FTO*) associated gene encoding the 2-oxoglutarate dependent DNA/RNA methylase interacts with lifestyle factors. The A allele of the *FTO* A > T (rs9939609) polymorphism in interaction with modifiable lifestyle factors has been associated with energy homeostasis, eating behavior, and appetite with an impact on body weight. Notably, some populations with European lineage have shown a higher risk allele frequency, thus predisposing them to extreme obesity (70).

Therefore, screening these genetic variants could be useful to identify genetically susceptible groups and prescribe tailored genome-based nutritional advice under a regionalized and personalized scope. On the other hand, the nutritional composition of the hepatopathogenic diet may lead to dyslipidemia (including hypertriglyceridemia), insulin resistance, oxidative stress, and the induction of a pro-inflammatory state, which jointly may contribute to the development of liver damage. In this context, a high prevalence of MASH and abnormal liver stiffness was reported in young Mexicans with obesity, even in normal-weight individuals consuming a high-fat/high-sugar diet (71). Altogether, these data reveal that among the admixed population of Mexico, the interaction between genes and lifestyle factors such as diet significantly impact liver health. Preventing the onset and progression of liver injury should be a priority by healthcare providers to avoid further disease. In this context, intervention studies have been conducted to avoid plausible diet-related chronic diseases. By using a genome-based nutrigenetic strategy, a Genomex diet was implemented to provide a resource for managing patients with a risk of chronic disease (72). This diet provided the appropriate recommended daily allowances of macro-, and micronutrients considering the staple foods and culture of the Mexican population and was based on the prevalence of some adaptive alleles that are predominant among the population. In this manner, patients who had altered metabolic parameters were normalized in 24 weeks with a significant reduction in body weight, BMI, insulin resistance, hypertriglyceridemia, and VLDL levels. Although this was a quasi-experimental study evaluating the effect on metabolic and clinical parameters before and after the Genomex intervention, a randomized trial evaluating the effect of a dietary intervention integrating some Mexican staples vs. a habitual diet on metabolic syndrome parameters yielded similar results. Only patients on the Mexican food-enriched diet decreased their triglyceride levels, glucose intolerance, and area under the curve for insulin, in addition, carriers of the *ABCA1* 230C variant showed greater weight loss and increased serum adiponectin (73).

### 4.2 Alcoholic liver disease

Alcoholic liver disease (ALD) is a chronic condition that requires a multidisciplinary approach involving medical, nutritional, and psychosocial interventions, which are key to avoiding disease recurrence and managing complications. Early intervention and adherence to treatment plans are crucial for improving outcomes in patients with ALD (74). The development of the disease is multifactorial, involving interactions between genetic susceptibility and lifestyle factors, such as alcohol consumption. Thus, PerMed-Nut strategies could be beneficial to tailor patient’s needs based on genetic and environmental factors.

Genes involved in alcoholism and the risk for chronic liver disease include taste receptors, neurotransmitter receptors, alcohol-metabolizing enzymes, and steatotic genes that are highly polymorphic worldwide (75, 76). Notably, some of these genes overlap with the risk for MAFLD and HCC. In a Mexican cohort, a 36% *TAS2R38* AVV haplotype homozygosity (non-taster phenotype) was found among drinkers compared to non-drinkers (61). Additionally, the *DRD2/ANKK1* Taq1A1 (rs1800497) polymorphism involved in addictive behaviors ranges up to 67% among the Amerindian subpopulations compared to 47% in the admixed groups (77). Likewise, a cohort of subjects displaying unhealthy food choices and altered biochemical parameters showed a high prevalence of this genetic variant (78). Thus, the riskier *TAS2R38/DRD2/ANKK1* genetic profile (affecting bitter taste perception and food reward, respectively) predisposes one to consume significantly more calories (alcohol or high-dense food), which may enhance hepatic damage. However, these associations need to be explored in other regions across Mexico and even in other populations worldwide.

Additionally, the genetic profile of the alcohol-oxidizing liver enzymes among the Mexicans reveals a high rate of the A1 allele (rs1229984) of the alcohol dehydrogenase 1B (*ALD1B*) gene, contrasting with a low rate of the protective A2 allele (rs671) of the aldehyde dehydrogenase (*ALDH2*) gene whereas the cytochrome P450 2E1 (*CYP2E1*) C2 allele (rs2031920) is highly present in the Amerindian populations (79). It is noteworthy to mention that despite a high-risk genetic profile tending toward liver damage, the peril will depend on both the specific combination of the risk or protective alleles (slow, intermediate, or fast metabolizers) and the pattern of alcohol consumption influenced by socio-cultural factors (75).

In this sense, the history of alcohol consumption among the Mexicans has evolved over the centuries in which distilled liquor was not regularly consumed by the Mesoamericans until the arrival of the Europeans. Most alcoholic beverages were fermented and obtained mainly from maize (tesgüino) or fruits (pulque from the agave plant), and mostly nobles (emperors or priests) had access to them. Only in circumstances such as illness o festivities were the laypeople allowed to consume alcoholic beverages. In contrast, the national alcohol consumption is currently an average of 4.4 L/*per capita* in young adults who begin to drink at early ages during the weekends despite public health warning measures, and eventually, alcohol intake increases (79). Thus, risk scores containing both the genetic and social factors involved in alcohol consumption should be taken into account to devise preventive strategies.

### 4.3 Hepatitis B infection

The first step in using PerMed-Nut strategies in the management of hepatitis B patients is to make an appropriate diagnosis. Personalized therapy is highly dependent on the patient’s genetic features and HBV genotype (80). HBV is divided into 10 HBV genotypes (A-J) based on the whole genome, each with a unique global distribution and clinical outcomes based on the interactions between the host and the virus (81). To date, five HBV genotypes may be significant for PerMed-Nut in Mexicans with different clinical implications. Among these, HBV genotypes H is predominant followed by G, F, A, and D which circulate in patients according to genetic and sociodemographic backgrounds (82, 83). Based on molecular epidemiology data, HBV genotypes A and D discovered in Mexico are deemed exotic when compared to the Americas’ prevalent H and F. Indeed, phylogenetic examination of those genotypes suggested that A2 originated in Europe and D4 in Africa. These findings are consistent with the demographic history of the Mexican population, as previously stated.

Regarding the clinical result, HBV genotypes A and D have been identified in individuals with chronic infection, but genotype H is common in occult hepatitis B cases and exhibits a high degree of adaptation among the local people (82). Interestingly, sub-genotype F1b native to South America and linked to HCC in Native Alaskans (84) and Native Peruvians (85) has been found in Mexican patients (86). However, the potential association of liver cancer in patients acutely infected with F1b has not been tested due to its low incidence (87). Nonetheless, among HIV or men who have sex with men (MSM) individuals, the presence of three HBV genotypes (in various combinations) increased the likelihood of severe liver fibrosis by 15-fold compared to dual-mixtures or single HBV genotype infections (88). As a result, in these populations, early diagnosis of the HBV genotype may help determine the risk of liver injury. Likewise, chronic hepatitis B patients often remain asymptomatic until decompensation, making late diagnosis of cirrhosis a challenge (89). Genetic markers could improve early diagnosis and prevent end-stage liver disease. Genome-wide association (Figure 2) and SNP studies reveal associations with clinical outcomes (90).

Pharmacological treatment is the primary target for managing chronic liver disease, considering mutations in the reverse transcriptase domain of the polymerase. The use of lamivudine should be avoided in samples with M204V/I or L180M + M204V mutations, and tenofovir should be monitored when quadruple mutation CYEI is detected (91). The main mutations associated with resistance to adefovir are A181T/V/S and N236T (92). Also, response to long-term lamivudine may be decreased in patients with genotype A since it is more susceptible to YMDD motif mutations (93).

Together with pharmacological treatment, adjuvant nutritional support, including vitamins and bioactive components like resveratrol, vitamin E, lactoferrin, selenium, curcumin, luteolin-7-O-glucoside, moringa extracts, chlorogenic acid, and epigallocatechin-3-gallate showing *in vitro* and *in vivo* anti-HBV activity may be beneficial for managing chronic hepatitis B patients. These nutrients are accessible and found in native American foods (94). The effectiveness of nutritional therapy will depend on the patient’s capacity to absorb anti-HBV nutrients, HBV genotype, and population ancestry.

### 4.4 Hepatitis C infection

#### 4.4.1 Immune response

PerMed-Nut strategies should include early detection of patients at risk of chronic HCV infection. Next-generation sequencing-based studies have identified human variants that predispose to susceptibility or are associated with self-resolution (95, 96). Some genetic markers are associated with HCV infection outcome, as they influence the immune response and lipid metabolic pathways. The interleukin 28-B (*IFNL3*) gene belonging to the type III IFN-β family is one of the best predictors of HCV infection outcome and induces antiviral and antitumor states through innate and adaptive immune responses (97, 98). The polymorphism, rs12979860 C/T, is located 3 kb upstream of the *IFNL3* gene. Patients homozygous for the CC genotype are more likely to achieve a sustained virological response (SVR) following a PEG-IFN-β plus ribavirin than CT and TT genotypes (99). The rs12979860-CC genotype, associated with spontaneous clearance in white, African, and Hispanic HCV patients, was found to be proportional to the SVR rate across ethnic groups (100, 101). In Mexico, the C allele has a frequency of 56.5% in admixed patients and was associated with self-clearance and less liver damage (101). Similarly, a polymorphism in the *IFNL4* rs368234815 (TT) gene was also associated with HCV clearance in Mexican mestizo patients. When these polymorphisms were analyzed in haplotype with the *IFNL3* rs8099917 SNP, the beneficial haplotype C/T/TT (rs12979860, rs8099917, and rs368234815) was associated with spontaneous clearance and less liver damage in Mexican patients (101).

#### 4.4.2 Lipid metabolism

APOE has several ligands in the hepatocyte, including low-density lipoprotein receptor (LDLR), syndecan-1 and syndecan-2, and heparan sulfate proteoglycans (102). APOE is also a structural component of the HCV envelope and mediates virion entry, assembly, and production (62). The *E4* allele is protective against HCV, as carriers have a higher chance of spontaneous recovery after infection and treatment with interferon plus ribavirin (103, 104). The *E2* allele protects against chronic infection, while the *E3* is associated with chronic infection (105). In Mexico, the *E4* allele has been associated with spontaneous clearance and less liver damage, while *E3* was associated with advanced fibrosis in chronic patients, which is present in 85% of the mestizo population (72), however, the *E2* allele exerted no effect (106). Therefore, there is a higher risk of developing advanced liver damage induced by HCV.

Clustering differentiation 36 (CD36) is a candidate receptor involved in the gustatory detection of lipids. Evidence suggests that genetic variation in the *CD36* gene can modulate the uptake of fatty acids. A promoter polymorphism -31118G > A, rs1761667 was studied among admixed Mexican HCV patients. It was demonstrated that the AA genotype had higher values of total fat and saturated fatty acids than non-GG genotypes. Additionally, AA genotype increased AST and liver fibrosis among chronic HCV-infected patients (59). It is known that the AA genotype decreases fat taste perception, thus, AA carriers could need an increased amount of fat to reach satiety. Thus, triggering the onset of fat liver accumulation that in conjunction with HCV infection potentializes liver damage among HCV patients.

The low-density lipoprotein receptor (LDLR) is a protein involved in the trafficking of lipoproteins containing APOE, such as VLDL and chylomicrons (107, 108). The rs688 C/T polymorphism protects against infection development, affects cholesterol levels and mRNA splicing, and decreases receptor surface expression levels. This makes LDLR less capable of lipid uptake, making it less susceptible to HCV.

As mentioned above, nutrition also plays a crucial role in HCV infection because it can modify serum lipid components and provide anti-HCV micronutrients. *In vitro* studies have shown the effect of certain nutrients with anti-HCV properties on the outcome of HCV infection, but no potential diet intervention has been performed in patients (55). A recent study in Mexican patients revealed that adherence to a fish-rich diet, mainly consisting of fish, seafood, and vegetable oils, had low viral loads and significant consumption of PUFAs ≥4.9% (109). Therefore, diets rich in anti-HCV macro- and micronutrients may affect HCV infection outcomes. Further dietary interventions are needed to clarify the role of diet in the management of HCV infection.

### 4.5 Hepatocellular carcinoma

Hepatocellular carcinoma seen as the end-stage outcome of chronic liver disease is influenced by various gene–environment interactions that vary depending on the population (81). In Mexico, HCC is low in prevalence which may be due to the population’s immune response to the HBV/H genotype, despite the high rate of occult HBV infection (82). Nonetheless, the influence of the emergent F1b, A, and D genotypes on the natural history of HCC has not been thoroughly studied (86).

Genetic variations involved in the development of HCC are very diverse, and each example correlates with specific susceptibility. The aforementioned *PNPLA3* I148M allele associated with MAFLD/MASH has been linked to the development of HCC in patients with obesity (65, 110). In Europe, individuals with the *PNPLA3* GG genotype, particularly those with severe obesity, have a higher risk of developing HCC (111, 112). In Japan, patients with obesity/MAFLD or chronic hepatitis C infection have a higher frequency of the GG genotype, making them more susceptible to HCC (113, 114). Furthermore, association of rs738409 *PNPLA3* and rs58542926 *TM6SF2* as a risk factor for HCC was reported in patients with different liver disease etiologies (115), suggesting that underlying altered lipid metabolism influences the outcome of chronic liver disease.

The Membrane-bound O-acyltransferase domain containing 7 (MBOAT7), also known as lysophosphatidylinositol acyltransferase 1 is involved in the remodeling phospholipid chains and controlling cell membrane desaturation (116). The reduced expression of the *MBOAT7* rs641738 T allele is associated with altered cell membranes and plasma composition, including cell fat accumulation, pro-inflammatory environment, MAFLD, MASH, severe fibrosis, and HCC (117–121). An Italian cohort showed an 80% increased risk of HCC due to the presence of the *MBOAT7* rs641738 T allele, contributing to the evolution of liver disease (118). However, the rs641738 T allele was not associated neither with HCC risk nor HBV infection in Chinese patients. (122).

Likewise, the hydroxysteroid 17-Beta dehydrogenase 13 protein encoded by the *HSD17B13* gene is involved in the metabolism of steroid hormones, prostaglandins, lipids, xenobiotics, and retinoids (123). This liver lipid droplet-associated enzyme is markedly upregulated in patients with MAFLD (124). HSD17B13 expression has been linked to steatosis, MASH, type 2 diabetes, and liver cancer (125) and was shown to be downregulated in an HCC model (126). Some *HSD17B13* variants such as the rs72613567 T > A have been associated with a protective effect in cirrhotic and HCC patients (127) as well as in European patients with ALD (128, 129) and HCV patients (130). In a recent study, this polymorphism was detected in patients with extreme obesity and MASH among other gen variants related to HCC (131). However, further genetic studies are also needed for LATAM populations, including Mexico, to confirm these associations.

Finally, a healthy diet can be potentially protective against HCC. Recent studies have identified nutrients, dietary patterns, and food groups with reduced, neutral, and high risk of developing HCC (132, 133). The nutrients reducing the risk of HCC are monounsaturated fatty acids, vitamin E, vitamin B9, beta-carotene, manganese, and potassium. Conversely, sodium, processed red meat, and sugar-sweetened beverages increase the risk. Currently, Mexicans consume a hepatopathogenic diet (48) with a potentially high risk of developing MAFLD/MASH setting the scenario that HCC may be underdiagnosed or increase in the future years (71, 134). The Genomex diet, containing a high content of protective nutrients has been implemented to prevent developing chronic liver disease (72). Furthermore, it has been reported that the treatment of alkaline-brined corn dough used to make the widely consumed Mexican tortillas can potentially protect against aflatoxin-induced HCC (135). However, further studies are needed to validate the role of diet in patients at risk of HCC, given the current prevalence of overweight and obesity.

## 5 Genomic education

Medical specialties and subspecialties, in their first stage of scientific medicine focused more on the complications of chronic disease, prolonging the patients’ life and improving their quality of life, but do not prevent in all cases disease remission (136). Faced with this situation, PerMed-Nut approaches based on genomic medicine/nutrition need to integrate medical, nutritional, sociocultural, and emotional/spiritual aspects of health (137). However, new medical education and training for medical professionals are required (138, 139). In LATAM and Mexico, updating the medical and other health sciences education curriculum both at the undergraduate and graduate levels must be considered a priority. This curriculum needs to integrate the so-called basic subjects: Biochemistry, Cell Biology, Genetics/Genomics, and Molecular Biology at different levels with bioinformatics, technological/digital strategies (Telemedicine) and novel clinical approaches focused on preventing chronic diseases and long-term complications (140). These basic subjects can be integrated sequentially during the first years of schooling, not independently, as in the past. In the last century, most medical specialties and careers in Nutrition were created separately. However, with this new understanding, physicians/clinicians need to integrate nutrition, while the nutritionist should handle the knowledge of disease the same way as the doctors work (141).

Likewise, hepatology was not born when most medical specialties were created (142). Although some hepatology postgraduate courses have been sponsored by the associations for the study of the liver to update medical specialists, pre-graduate doctors and nutritionists need to understand the molecular physiology of the liver, while more postgraduate courses in MedPer-Nut are required to advance in Genomic Hepatology (143). Furthermore, the capability to analyze hundreds or thousands of these genes simultaneously leads us to large-scale data management (Big Data) and specialized bioinformatics (machine learning and other artificial intelligence methods), together with advanced omics biotechnologies (epigenetics, metagenomics, proteomics, lipidomics, and metabolomics), digital media, and electronic medical records (144). These applications are not the Medicine of the future, they are the present Medicine that will require training and re-constructing health career curriculums.

**Figure 4:**
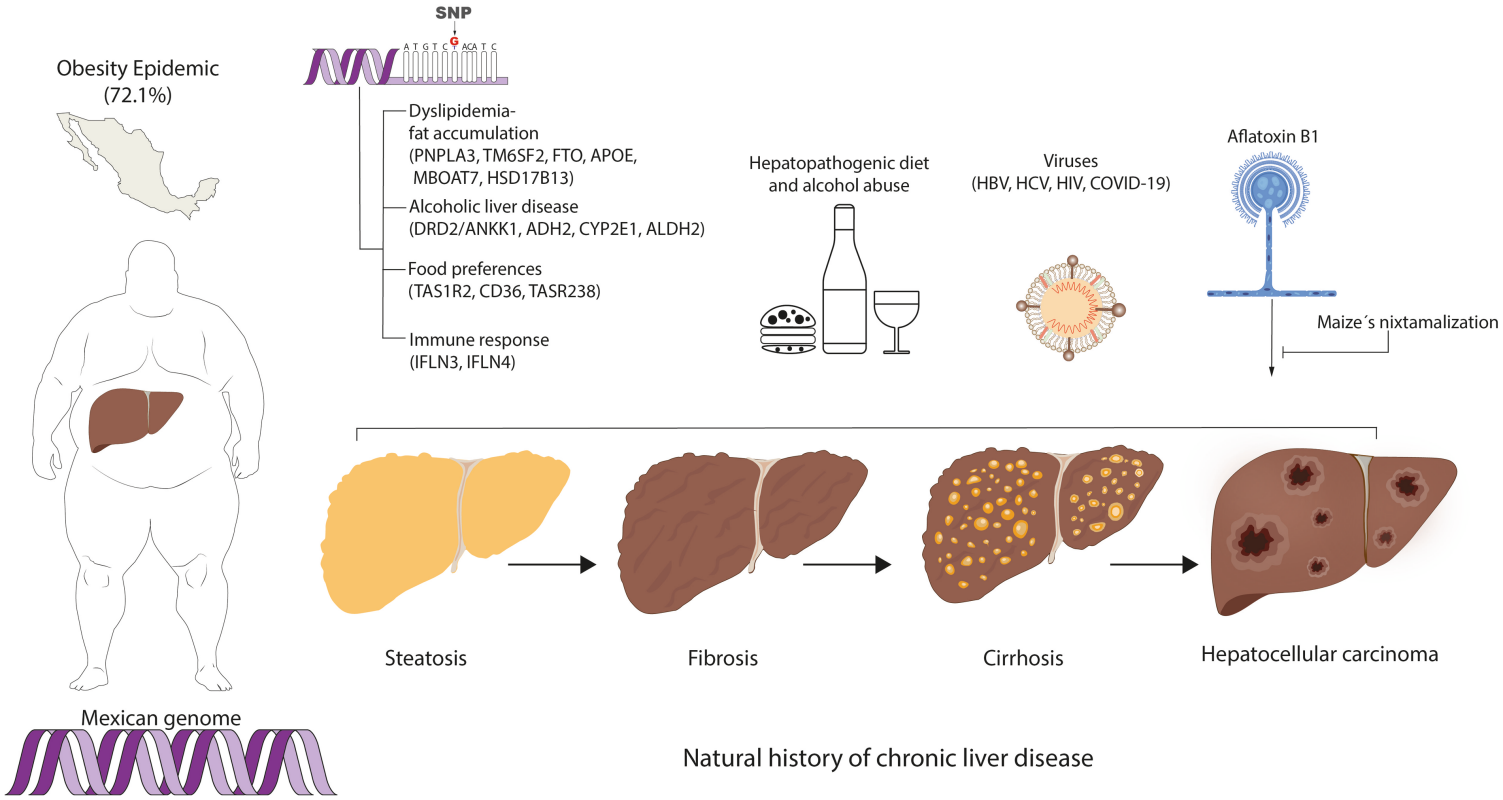
Personalized medicine and nutrition in hepatology among the Mexican population. The concurring genetic and environmental factors involved in chronic liver disease among the Mexican population are components to consider for the implementation of regionalized prevention strategies for chronic liver disease.

Lastly, the major benefit of re-engineering the educational curriculum is to promote the formation of researchers in LATAM and Mexico, thus creating novel knowledge in the field of MedPer-Nut in this region (145). In this sense, transforming the present clinical practice guidelines is an ongoing challenge because most recommendations are based on studies carried out in foreign populations (146). As illustrated in Figure 4, the genetic profile interacting with the environmental factors that concur in Mexico, may not replicate in other regions and vice versa. Thus, the vision is to prevent the onset and progression of chronic liver disease using MedPer-Nut approaches concordant with the genetic and environmental characteristics of the population (147).

## 6 Discussion

In the field of Medicine, we are now moving toward a new landscape of what exactly health means and how the pathological process begins. A primary goal is to avoid the onset of chronic diseases such as obesity, type 2 diabetes, and cardiovascular and liver diseases with their associated complications in the advanced stages. Reaching this goal will require detecting the genetic susceptibility to such diseases at young ages or at early stages, avoiding the pathology’s development as is the case of liver cirrhosis due to alcohol, viral hepatitis, or MASLD. Such studies are the beginning of the pathway toward the new age of PerMed-Nut in liver diseases.

In perspective, 20 years ago, it was commonly said that genes and environment interact with each other, but it was not clear how such interactions took place, nor which were the main environmental factors. Currently, it is recognized that at least three environmental factors constantly interact with our genes: diet (micro and macronutrients), physical activity (exercise), and emotions (stressors) (137). Human adaptability to these elements lies in the genetic variations gained through evolution that mark the differences between individuals or populations at the genomic and cultural levels (47, 148, 149). As shown in this review, within LATAM, despite having a common social history of the peopling of the continent, a wide spectrum of ancestral inter-variability is notable as well a distinct environmental conditions (24, 25, 28, 33). In the case of Mexico, it has been shown that the North–South gradient of the European-Amerindian ancestry impacts importantly in the distribution of the several genes with biomedical implications for the risk of chronic liver diseases (Tables 2, 3; Figure 3) (32, 44, 45, 153). More so, this pattern of distribution could also be replicated intra-regionally in light of the social-demographic movements that occurred over time as in the case of the *ABCA1* polymorphism (46). This feature is the reason for further studies regarding the plausible association of certain genetic alleles with the clinical outcomes to tailor preventive strategies.

**Table 3:** Summary of genetic variants related to etiologies of liver conditions or disease based on ancestry.

Gene	Polymorphism	Risk/Protective allele	Nutritional/Clinical association	Statistical value^*^	Ancestry^**^	Reference
*Metabolic-associated steatotic liver disease*						
*CD36*	−31118 G>A rs1761667	A	Higher fat intake and hypercholesterolemia	2.75 (1.33–2.75) *p* = 0.005	Admixed Mexican	(58)
	*TAS1R2*			Ile191Val rs35874116			Val	High carbohydrate intake and hypertriglyceridemia	2.61 (1.12–6.07) *p* = 0.02	Admixed Mexican	(60)
*APOE*		rs429358/rs7412	E4	High prevalence of hypercholesterolemia	*p* = 0.047	Amerindian			(53)		
	E2		High prevalence of hypertriglyceridemia	*p* = 0.045	Admixed Mexican				
*PNPLA3*	Ile148Met (C > G) rs738409	Met	High ALT serum levels	3.7 (2.3–5.9) *p* = 3.7 × 10^−8^	Amerindian Admixed Mexican	(68)
	*SIK3*			G > A rs139961185	A		High triglycerides levels after a high-fat meal	1.44 (1.27–1.63) *p* = 1.15 ×10^−12^	Admixed Mexican	(150)
*SIDT2*		G > A rs17120425	A	High HDL-C levels		2.92 (0.98) *p* = 0.005		Admixed Mexican	(151)	
	*APOA5*	G > C rs964184			G	High triglycerides levels	*p* = 5.32×10^−37^ BETA = 0.289	Admixed Mexican		(152)
*Alcoholic liver disease*										
*TAS2R38*	A49P rs13598	AVV haplotype	High consumption of alcohol	1.79 (1.13–2.84) *p* < 0.05	Admixed Mexican	(61)
	V262A rs1726866					
	I296V rs10246939					
*DRD2/ANKK1*	Taq1A A1 > A2 rs1800497	A1	High consumption of alcohol	4.09 (1.56–10.68) *p* = 0.0021	Admixed Mexican	(77)
				*ADH1B*			Arg48His rs1229984	Arg (A1)	Low frequency of protective allele (A2)	ND	Admixed	(79)
Amerindian												
*ALDH2*	Glu504Lys rs671	Glu (A1)	Low frequency of protective allele (A2)	ND	Amerindian							
*CYP2E1*	−1055C > T rs2031920	T (C2)	High frequency of risk allele	ND	Amerindian							
*Hepatitis C infection*						
*IFLN3*	rs12979860/	C/T/TT haplotype	Spontaneous clearance and less liver damage	0.46 (0.22–0.95) *p* = 0.03 and 0.32 (0.10–0.97), *p* = 0.04	Admixed Mexican	(101)
*IFLN4*	rs8099917/rs368234815					
	*APOE*						rs429358/rs7412	E4	Spontaneous clearance and less liver damage	0.55 (0.31–0.98) *p* = 0.042 and 0.091 (0.01–0.75) *p* = 0.020	Admixed Mexican	(106)
E3		Severe fibrosis (F3-F4)	2.99 (1.13–7.87) *p* = 0.02	Admixed Mexican								
*CD36*	−31118 G > A rs1761667	A	High-fat intake, serum AST values, and advanced liver fibrosis	3.60 (1.16–11.15) *p* = 0.02	Admixed Mexican	(58)
*Hepatocellular carcinoma*						
*PNPLA3*	Ile148Met	Met	Development of HCC in patients with obesity, viral infection, and ALD	5.88 (1–45–23.80) *p* = 0.013	Caucasian	(110)
				2.62 (1.15–5.96) *p* = 0.0218	Japanese	(114)
				3.91 (2.52–6.06) *p* = 1.14 × 10^−9^	Caucasian	(115)
*MBOAT7*	rs641738	T	HCC risk	2.18 (1.30–3.63) *p* = 0.003	Caucasian	(118)
			Liver fat	0.034 (0.018–0.051) *p* = 4.8 × 10^−5^		(121)
			Fibrosis	1.21 (1.03–1.45) *p* = 0.021		
*HSD17B13*	rs72613567	A	Reduced HCC risk in HCV and ALD	0.71 (0.60–0.85) *p* = 0.002	Caucasian	(128)
				0.73 (0.65–0.82) *p* ≤ 0.0001		

Source: Data extracted from the indicated references. ^*^Figures are OR (95% CI), *p* value or *p* value alone when compared to opposing genotypes/alleles or study group. ^**^Data regarding hepatocellular carcinoma and specified genes has not been studied in Mexican population. ND, not determined.

The diet-related alleles that have been studied to date among the Mexican population are key elements in the development of chronic illnesses including liver disease. As shown in Table 3, some polymorphisms are related to high food preferences for carbohydrates or lipids, altered food behaviors (excessive alcohol consumption), and dyslipidemias that can partly explain the increasing onset of MAFLD among the Mexican population. Interestingly, several genomic studies in Mexican Amerindian and admixed cohorts have identified the signature of positive selection of some serum lipid-modulating gene traits involved in cardiovascular diseases (150–152) that plausibly could overlap with the natural history of MAFLD. Overall, these genes can be useful for the development and validation of polygenic risk scores testing for the susceptibility in liver diseases among the Mexican population in the context of the tripartite ancestry (154–156). On the other hand, the impact of nutrition in patients with chronic viral hepatitis, specifically hepatitis B and C is an opportunity of exploration since patients with these pathologies have unmet needs in terms of their nutrition care to prevent further liver damage due to genetic susceptibility and harmful eating habits. Ultimately, understanding the molecular basis of these pathologies and the interactions that take place with the environment will derive in the making of a PerMed-Nut strategy appropriate for the Mexican population focused on the implementation of preventive strategies based on nutrition, exercise, and mental health instead of treating advanced diseases (157).

## 7 Conclusion

LATAM including Mexico shares a common ancestry in terms of genetics and culture that have an impact on health. However, further research regarding the distribution of genetic risk alleles and the interaction with environmental factors is required in this region. PerMed-Nut strategies based on these factors are the forthcoming trend in the field of Genomic Hepatology and other fields of medical specialties for preventing and managing chronic liver diseases. Knowledge of the genetic and lifestyle factors involved in the onset and progression of the major etiologies of chronic liver diseases needs to be generated by local researchers to provide regional clinical practice guidelines concordant with the features of the population. Achieving this PerMed-Nut approach is not without challenges since updated Medicine and Nutrition education curriculums are required. Training and preparing future health professionals and researchers with new clinical and investigative skills focused on preventing liver diseases in the field of Genomic Hepatology globally is a vision that clinicians and nutritionists should be concerned about.

## Author contributions

AP: Conceptualization, Investigation, Visualization, Writing – original draft, Writing – review & editing. SR: Conceptualization, Investigation, Visualization, Writing – original draft, Writing – review & editing. IM-M: Investigation, Visualization, Writing – review & editing. AJ-A: Investigation, Visualization, Writing – review & editing. KG-A: Investigation, Visualization, Writing – review & editing. CO-G: Investigation, Visualization, Writing – review & editing. OR-L: Investigation, Visualization, Writing – review & editing. LT-R: Investigation, Visualization, Writing – review & editing.
